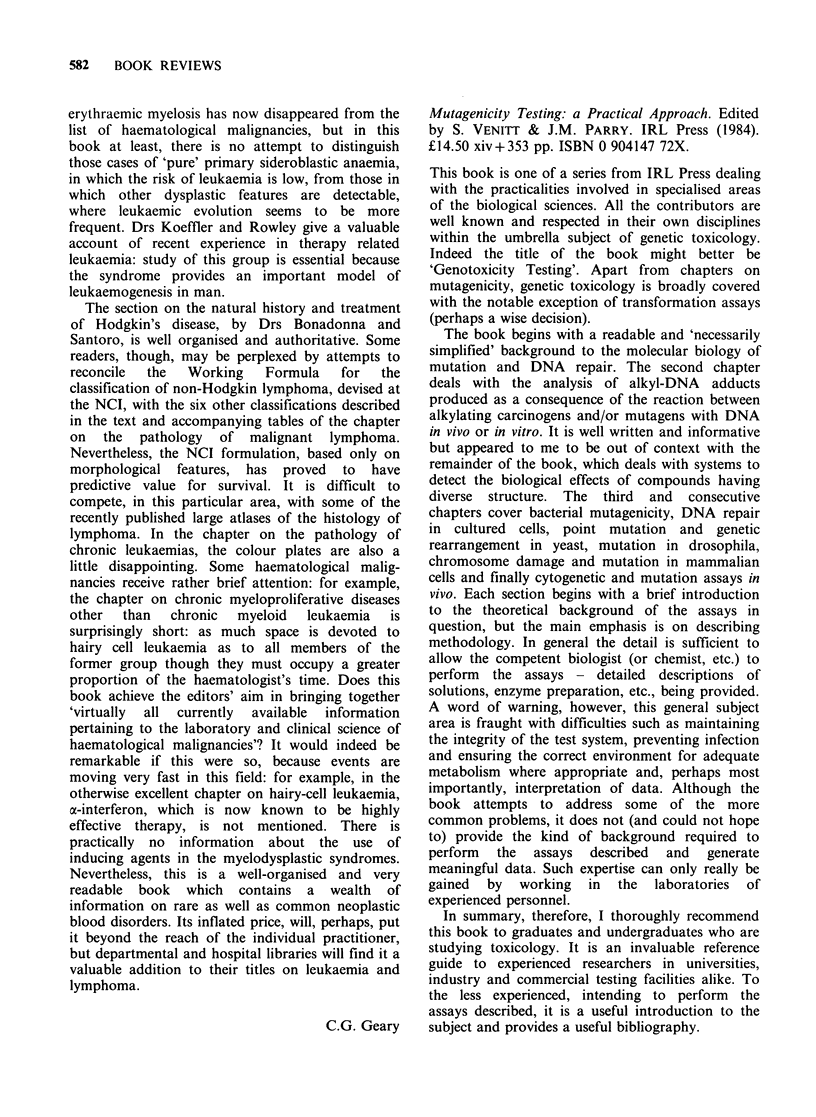# Mutagenicity Testing: a Practical Approach

**Published:** 1986-04

**Authors:** 


*Mutagenicity Testing: a Practical Approach.* Edited by S. Venitt & J.M. Parry. IRL Press (1984). £14.50 xiv+353 pp. ISBN 0 904147 72X.

This book is one of a series from IRL Press dealing with the practicalities involved in specialised areas of the biological sciences. All the contributors are well known and respected in their own disciplines within the umbrella subject of genetic toxicology. Indeed the title of the book might better be 'Genotoxicity Testing'. Apart from chapters on mutagenicity, genetic toxicology is broadly covered with the notable exception of transformation assays (perhaps a wise decision).

The book begins with a readable and 'necessarily simplified' background to the molecular biology of mutation and DNA repair. The second chapter deals with the analysis of alkyl-DNA adducts produced as a consequence of the reaction between alkylating carcinogens and/or mutagens with DNA *in vivo* or *in vitro*. It is well written and informative but appeared to me to be out of context with the remainder of the book, which deals with systems to detect the biological effects of compounds having diverse structure. The third and consecutive chapters cover bacterial mutagenicity, DNA repair in cultured cells, point mutation and genetic rearrangement in yeast, mutation in drosophila, chromosome damage and mutation in mammalian cells and finally cytogenetic and mutation assays *in vivo*. Each section begins with a brief introduction to the theoretical background of the assays in question, but the main emphasis is on describing methodology. In general the detail is sufficient to allow the competent biologist (or chemist, etc.) to perform the assays – detailed descriptions of solutions, enzyme preparation, etc., being provided. A word of warning, however, this general subject area is fraught with difficulties such as maintaining the integrity of the test system, preventing infection and ensuring the correct environment for adequate metabolism where appropriate and, perhaps most importantly, interpretation of data. Although the book attempts to address some of the more common problems, it does not (and could not hope to) provide the kind of background required to perform the assays described and generate meaningful data. Such expertise can only really be gained by working in the laboratories of experienced personnel.

In summary, therefore, I thoroughly recommend this book to graduates and undergraduates who are studying toxicology. It is an invaluable reference guide to experienced researchers in universities, industry and commercial testing facilities alike. To the less experienced, intending to perform the assays described, it is a useful introduction to the subject and provides a useful bibliography.